# Carpometacarpal Osteoarthritis Pain of the Thumb Can Be Relieved by Commercial Beverage Carbonated Water

**DOI:** 10.1155/2024/6238171

**Published:** 2024-02-24

**Authors:** Masaaki Nakajima

**Affiliations:** Department of Physical Therapy, School of Health Science and Social Welfare, Kibi International University, 8 Iga-Machi, Takahashi, Okayama 716-8508, Japan

## Abstract

Thumb carpometacarpal (CMC) arthropathy pain is treated using carbonated water—a commercially available beverage. The right hand (affected side) was bathed once daily in carbonated beverage water (37°C) for 20 min. Prior to treatment, the visual analogue scale score of pain was 73 mm; 1 week after the treatment, it was 0 mm. Commercial carbonated water immersion was effective for thumb CMC arthropathy pain. Commercial carbonated water is inexpensive and easy to obtain, making it suitable for home carbonation therapy.

## 1. Introduction

Thumb carpometacarpal (CMC) osteoarthritis is a degenerative condition of the joint at the base of the thumb that causes pain, stiffness, and weakness while performing movements that may require grip force of the thumb, such as picking up objects or opening bottle lids. Conservative treatment includes the administration of percutaneous antiphlogistic analgesics and the use of a brace such as soft orthosis to limit movement. Percutaneous antiphlogistic analgesics exert their anti-inflammatory effects by inhibiting the biosynthesis of prostaglandins, which are chemical mediators of inflammation.

Alternatively, CO_2_-enriched water bathing has an excellent effect on blood flow and is used to relieve fatigue, reduce pain, and promote wound healing [1–4]. I also created an experimental skin defect model using rats and confirmed the excellent wound healing promotion effect of CO_2_-enriched water bathing. A 3-cm-diameter skin defect was created on the back of 13-week-old Wistar rats, and they were subjected to 20-minute bathing of either 35°C fresh water or 35°C CO_2_-enriched water (CO_2_ concentration, 1000 ppm) five times a week. The number of days required for wound healing was 35.4 ± 0.9 days with fresh water bathing, whereas it was shortened to 23.8 ± 0.4 days with CO_2_-enriched water bathing.

In this study, I hypothesized that the application of CO_2_-enriched water treatment to thumb CMC arthropathy could alleviate pain by eliminating inflammatory chemical mediators.

Nowadays, artificial CO_2_-enriched water is generally prepared using artificial CO_2_-enriched water-making equipment [4]; however, only a few hospitals have such equipment. If one thinks about it, the therapeutic effect of CO_2_-enriched water treatment is brought about by the percutaneous penetration of CO_2_ dissolved in water into the body. Therefore, using commercial beverage carbonated water for drinking should yield the same effects.

In this short report, I present a case of thumb CMC arthropathy pain treated using carbonated water—a commercially available beverage.

## 2. Methods

**Figure 1:**
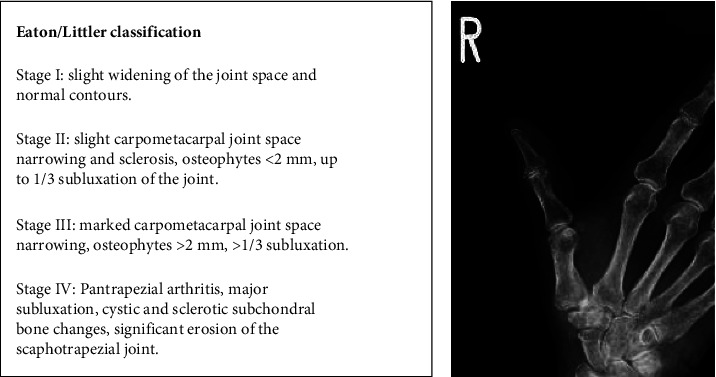
“Eaton/Littler classification” and the patient's X-ray image. (a) Eaton/littler classification classifies thumb carpometacarpal joint osteoarthritis into four grades based on radiographic images. (b) Narrowing of the joints is observed and classified as stage II.

Case: A 73-year-old obstetrician-gynecologist presented to an orthopedic surgeon with a complaint of pain on motion and tenderness in the right thumb CMC joint and was diagnosed with CMC osteoarthritis. X-ray images showed a narrowing of the joint space, and the severity of the disease was classified as stage II according to the Eaton–Littler classification [6] using X-rays (Figure 1). Despite the administration of anti-inflammatory analgesics and restriction of movement with a soft orthosis brace, the pain did not abate even after 6 months.

**Figure 2:**
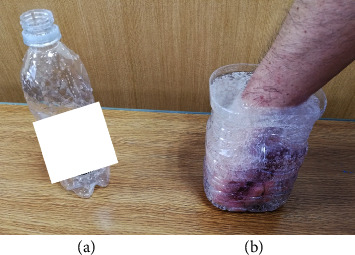
Treatment of thumb carpometacarpal arthropathy using the commercial beverage, carbonated water. (a) Commercial carbonated water (500 mL). (b) A container made from a 2 L plastic bottle cut in half and filled with carbonated water. The affected hand is immersed in the container in this position for 20 min. CO_2_ dissolved in water penetrates the skin percutaneously; therefore, bubbles present on the skin show impeded penetration of CO_2_. If there are too many bubbles, then gently remove the hand from the carbonated water, remove the bubbles, and reapply the soak.

Treatment: The right hand (affected side) was bathed once daily in CO_2_-enriched water for 20 min. Commercial carbonated water was used for this treatment (Figure 2). To exclude the effects of components other than carbonation, I chose commercial carbonated water without fruit juice or flavorings and opted for pure carbonated water. Initially, its temperature was approximately 37°C [7]. It was then heated indirectly by filling the basin with hot water and placing a polyethylene terephthalate bottle of commercial carbonated water in it.

Evaluation: The pain was evaluated using a visual analogue scale.

## 3. Result

Prior to treatment, the visual analogue scale score was 73 mm; 1 week after the treatment, it was 0 mm. Within 2 weeks, the treatment was completed, and the patient experienced no pain even after 3 months of stopping the treatment.

## 4. Discussion

The pain was relieved within 1 week of starting the 20-minute immersion therapy once daily in commercial carbonated water. These results support the hypothesis that CO_2_-enriched water treatment for pain during motion in the thumb CMC joint enhances blood flow, removes inflammatory chemical mediators, and relieves pain.

**Figure 3:**
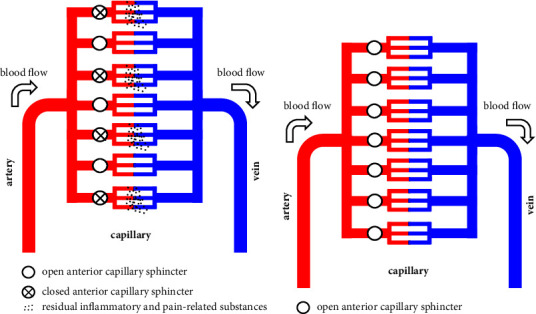
Blood flow in the capillary network in carbonated water immersion. Blood flow to the capillary network is regulated by the alternating contraction and relaxation of the anterior capillary sphincter. With a closed anterior capillary sphincter, massage or bathing to promote blood flow is unlikely to eliminate inflammatory chemical mediators (a). In a carbonated water bath, the anterior capillary sphincter muscles relax in all areas where percutaneous CO_2_ reaches, thus causing smooth muscle relaxation. Therefore, metabolites and inflammatory substances can be efficiently removed, and excellent muscle fatigue recovery and pain relief can be expected (b).

Inflammation results in the production of inflammatory mediators such as prostaglandins. Prostaglandins and other inflammatory chemical mediators lower the threshold of pain receptors. Therefore, the accumulation of inflammatory chemical mediators such as prostaglandins lowers the threshold of pain receptors and causes motor pain. Generally, analgesics exert their effects by inhibiting the production of inflammatory chemical mediators such as prostaglandins. In contrast, the pain-relieving effect of carbon dioxide baths is achieved by flushing out inflammatory chemical mediators by promoting blood flow through the relaxation of vascular smooth muscle [8]. Blood flow to tissue capillaries is regulated by the Autonomic Nervous System Regulation of the precapillary sphincter in front of it, and there is a network of capillaries in which some precapillary sphincters are closed and dormant. Therefore, heat therapy or massage to stimulate blood flow does not eliminate inflammatory chemical mediators in the capillary network of the annulus region of the closed anterior capillary sphincter. However, when the affected area is immersed in carbonated water, percutaneously introduced CO_2_ relaxes the anterior capillary sphincter. The anterior capillary sphincter, which had been closed by Autonomic Nervous System Regulation, also relaxes, stimulating blood flow in all capillary networks, eliminating inflammatory chemical mediators, and making the pain disappear (Figure 3). The mechanism of vascular smooth muscle relaxation by percutaneously entered CO_2_ has not been clarified, but it is thought to be due to inhibition of Ca^2+^ channel activity. CO_2_ that enters the body reacts quickly with water, mediated by carbonic anhydrase, to form bicarbonate and hydrogen ions. It has been shown that an acidic pH tilt of the extracellular fluid of vascular smooth muscle cells may inhibit Ca^2+^ channel activity and decrease contractility through a decrease in intracellular pH [9, 10]. Consider why the commercial beverage carbonated water bathing worked remarkably well for thumb CM arthritis pain. The first reason is that the CMC joints of the thumb have thinner tissues than the knee and hip joints, making it easier for percutaneous CO_2_ to reach the affected area, which is thought to be the cause of the high pain-relieving effect of the treatment. As mentioned above, when the affected area is immersed in carbonated water, the CO_2_ dissolved in the water percutaneously enters the body and exerts a vascular smooth muscle relaxant effect. In a living body, it is difficult for CO_2_ to reach deeper parts of the body because it is carried away by the blood flow in the process of penetrating deeper into the body. Therefore, the deeper the tissue in the body, the smaller the effect of vascular smooth muscle relaxation. The knee and hip joints are large, making it difficult for sufficient concentration of CO_2_ to reach the deep inflammatory zone. However, the thumb CMC joints are thin, and the high concentration of CO_2_ in the inflammatory chemical mediator reservoirs may have had a particularly strong flush-out effect. The second reason could be the high CO_2_ concentration in the commercial carbonated water. The effect of carbonated water bathing on blood flow is dependent on the CO_2_ concentration [11]. While the CO_2_ concentration of an artificial carbon dioxide spring is 1000 ppm, commercial beverage carbonated water is approximately 5000−7000 ppm [12], several times higher than the carbon dioxide concentration of artificial carbonated water. The superior pain-suppressing effect of immersion in a carbonated water bath for commercial beverages is thought to be due to the use of highly concentrated carbonated water, which has a higher blood flow-promoting effect than carbonated water bathing with a normal concentration.

Next, consider the fact that the patient no longer experienced pain after discontinuing the commercial beverage carbonated water bathing. Carbonated water bathing has been reported to increase natural killer cell (NK cell) activity [12]. NK cells are mainly located in the blood and are one of the immune cells among lymphocytes, which are lymphocytes that attack foreign or abnormal cells when they are detected. Increased NK cell activity means enhanced immunity, and carbonated water bathing is thought to promote calming of inflammatory symptoms.

Tissue pH is acidotic when carbon dioxide enters the tissues, and acidosis promotes the expression of VEGF and bFGF [13, 14]. VEGF is a growth factor involved in angiogenesis and stimulates cell division, migration, and differentiation and is also involved in the activation of monocytes and macrophages. FGF is a type of growth factor related to angiogenesis and wound healing and plays an important role in the process of cell and tissue proliferation and differentiation. Expression of VEGF and bFGF by carbonated water bathing may enhance immunocompetence, wound healing, and promote the calming of inflammation. Although there is concern that angiogenesis may worsen inflammation in inflamed tissues, carbonic acid baths suppress angiogenesis despite the increased expression of VEGF and bFGF [12], suggesting that there are no negative effects.

The vasodilatation caused by the carbonated water bathing may have improved inflammation by increasing the presence of nutrients, oxygen, and other materials for cell regeneration and by enhancing the immune response and promoting wound healing.

Use of Commercial Beverage Carbonated Water for Carbonated Bath Therapy: In recent years, CO_2_-enriched water treatments for pain relief, fatigue recovery, and wound healing have been performed using artificial CO_2_-enriched water preparation equipment; however, few hospitals have such equipment [4]. However, commercial beverage carbonated water is inexpensive and easy to obtain; thus, the treatment can be performed at home. The CO_2_ concentration is 5000–7000 ppm, which is higher than that of artificial carbonated springs (1000 ppm), and thus can be expected to have an excellent effect on promoting blood flow. In cases where the treated area is small, such as in CM arthritis of the thumb, a small amount of carbonated water may be required, making carbonated water bathing with commercial beverage carbonated water suitable. This insight suggests the potential use of commercially available carbonated water for carbonated spring bath therapy, addressing the issue of preparing carbonated water for carbonated spring therapy. It is considered valuable information that enables carbonated water therapy at home.

### 4.1. Limitations of the Study

This research is a report of a single case. The possibility of placebo effects and natural resolution of symptoms cannot be ruled out. More reports from a larger number of cases will be needed in the future.

## 5. Conclusion

Our study concluded that immersion of the affected hand in commercial beverage carbonated water once a day for 20 min is effective in treating pain caused by thumb CMC osteoarthritis. The high concentration of CO_2_ easily reaches the inflamed area in osteoarthritis of the thumb, and the high CO_2_ concentration in commercial beverage carbonated water has a high flush-out effect on pain-related substances and is considered to have an excellent analgesic effect. Commercial carbonated water is inexpensive and easy to obtain, making it suitable for home carbonation therapy if the treated area is small. This study is a single case, and a randomized controlled trial is needed to substantiate this finding.
